# Shared decision making for perioperative antibiotic use during Mohs micrographic surgery on the lower extremities

**DOI:** 10.1016/j.jdin.2024.03.011

**Published:** 2024-03-28

**Authors:** Lisa Fronek, Michael J. Davis, Hubert T. Greenway, Benjamin Kelley

**Affiliations:** a Department of Dermatology, Scripps Clinic Torrey Pines Bighorn Mohs Surgery and Dermatology, San Diego, California; b Department of Dermatology, Memorial Sloan Kettering, Mohs Micrographic Surgery, New York, New York

**Keywords:** antibiotic prophylaxis, Mohs surgery, postoperative skin infection, shared decision making

## Abstract

**Background:**

While there is a higher risk of surgical site infection (SSI) on the lower extremities following Mohs micrographic surgery (MMS), antibiotic prophylaxis (AP) is debated.

**Objective:**

To determine the role of shared decision making (SDM) in guiding AP usage during MMS on the lower extremities.

**Materials and methods:**

A prospective observational study was conducted whereby patients received a standardized SDM discussion or routine counseling. Patient satisfaction quantified by the shared decision-making questionnaire (SDMQ9) survey, rate of SSI, and rate of AP prescription were recorded.

**Results:**

In total, 51 patients were included. While there were less antibiotics prescribed in the treatment group (20% versus 50%, *P* = .025), this did not affect incidence of SSI (8% in treatment group versus 7.7% in control group, *P* = .668). Patient satisfaction was statistically greater in SDM group (4.73 versus 2.18 in control (*P* < .001).

**Conclusion:**

Patient satisfaction scores were higher among the patients who received SDM. While the usage of AP was lower in the SDM group, this did not affect incidence of SSI. This study allows the opportunity to apply SDM in the setting of MMS, which to our knowledge has not yet been attempted in the field of dermatologic surgery.

**Capsule Summary**

• This article expands on the role of antibiotic prophylaxis in reducing postoperative superficial skin infection following Mohs micrographic surgery on the lower extremities.
• The data presented make a strong case for Mohs surgeons to incorporate Shared Decision Making into practice to facilitate a discussion about the role of antibiotic prophylaxis.

## Introduction

Mohs micrographic surgery (MMS) is an office-based procedure performed under either sterile or clean technique.^1^^,^^2^ While MMS has a low rate of adverse events, the most frequent complication is a surgical site infection (SSI).^1^^,^^3^ The overall risk of SSI following MMS ranges from 0.7% to 2.5%^4^^,^^5^; this risk approaches 6.9% to 7.5% on the lower extremities.^6^^,^^7^ Surgical site infection is associated with increased morbidity, poor wound healing, and poor cosmetic outcomes.^3^^,^^8^ Additionally, SSIs create a burden to the patient and the healthcare system.^4^^,^^8^ Many clinicians recommend antibiotic prophylaxis (AP) either pre-/intra- or postoperatively, despite contradicting evidence about whether AP decreases the risk of SSI following MMS on the lower extremities.^1^^,^^3^^,^^6^^,^^9^, ^10^, ^11^, ^12^ While an advisory statement in 2008 and review of literature recommended AP for MMS on the lower extremities, these recommendations are largely anecdotal.^4^^,^^13^ Several studies have documented the lack of risk reduction of SSI when employing AP.^1^^,^^6^^,^^11^

Despite the lack of evidence, many MMS surgeons continue to utilize oral AP on the lower extremities.^14^^,^^15^ A survey of the American College of Mohs Surgeons found that 8.1% of respondents give a preoperative loading dose of AP and 33.6% of respondents utilize a 7-to-14-day course of AP for MMS on the lower extremities.^16^ Physicians should be cognizant about AP usage in the context of rising rates of Methicillin-resistant Staphylococcus aureus and the morbidity associated with adverse effects of oral antibiotics.^4^^,^^16^ Shared decision-making (SDM) is the process whereby patients and their physicians discuss management options and decide together how to proceed.^17^^,^^18^ Many studies have found SDM to be useful in educating patients as well as fostering a healthy relationship between physicians and their patients.^19^^,^^20^ A recent review of SDM use in dermatology found SDM to be a beneficial tool in providing patient-focused care.^19^, ^20^, ^21^

## Objective

**Fig 1:**
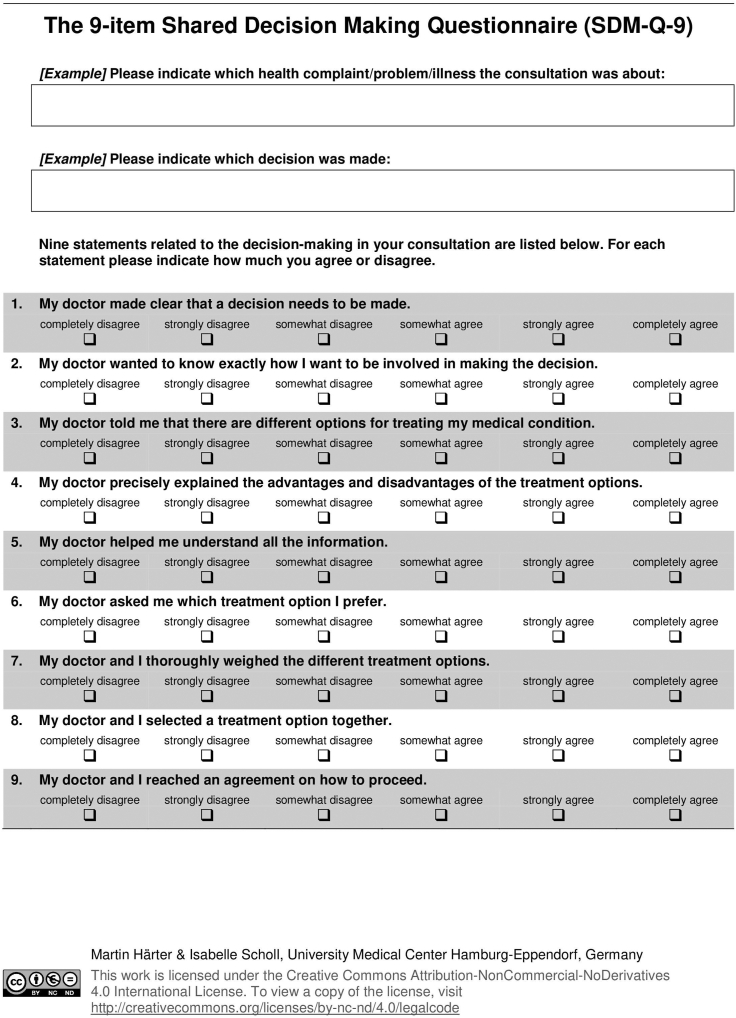
The Shared Decision-Making Questionnaire-9 is a verified, standardized 9-item self-assessment that quantifies the patients’ perception of their role in decision making.

This study utilized an SDM pamphlet to educate patients about their risk of SSI following MMS, the lack of evidence of risk reduction with AP, and the various management options available. The aim of this study is to determine the role of SDM in guiding use of AP during MMS on the lower extremities. The primary outcome of this study is patient satisfaction as measured by the 9-item Shared Decision-Making Questionnaire (SDMQ9) (Fig 1).^22^ The secondary outcomes include the difference in rates of postoperative SSI and AP selection between the treatment and placebo groups.

## Methods

### Demographics

There were 51 patients, aged 18 or older assessed in this prospective observational study at Scripps Clinic Torrey Pines Mohs Surgery Center from October 1, 2022 to February 14, 2023. All 51 patients had a biopsy proven basal cell carcinoma (BCC) or squamous cell carcinoma (SCC) on a lower extremity, below the knee, excluding the foot. Patients were excluded if they had co-existing diabetes mellitus (DM), peripheral vascular disease, tobacco use, prosthetic joint replacement, heart valve replacement, rheumatic fever, pacemaker/defibrillator etc, or if the patient underwent a flap, full thickness skin graft, or split thickness skin graft for repair.

**Fig 2:**
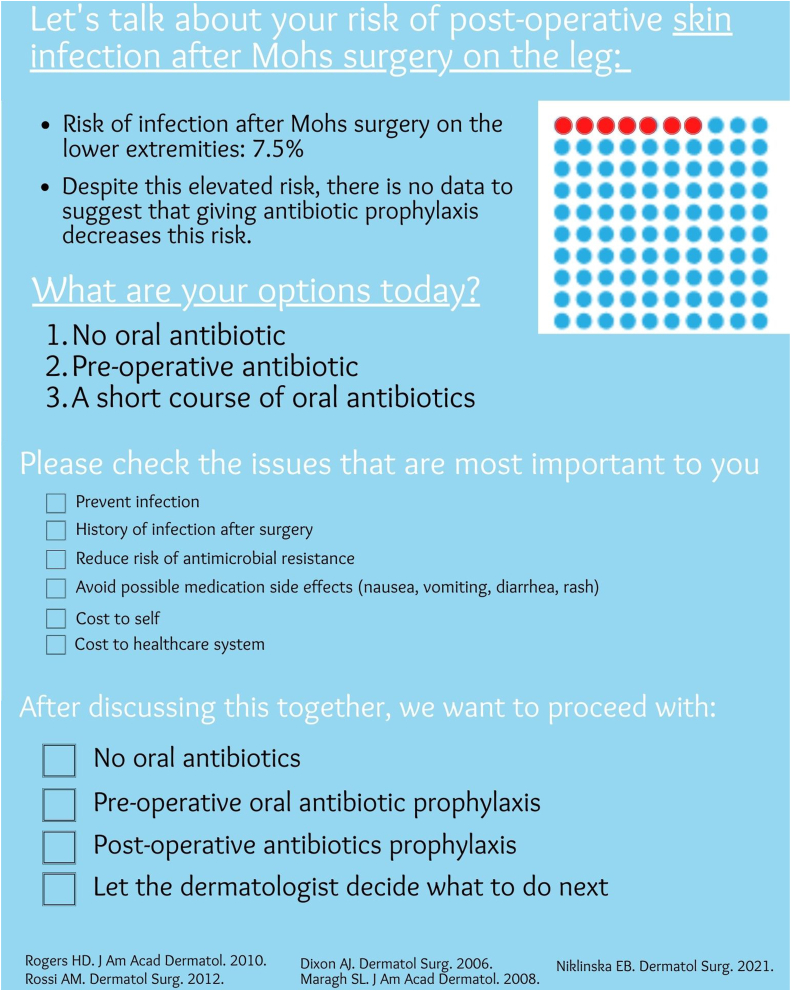
Shared Decision-Making pamphlet created by Lisa Fronek D.O., revised by Benjamin Kelley M.D. and approved by Hubert Greenway M.D.

Written informed consent was obtained and patients were randomized into 2 groups using a randomized computerized generator. Half of the patients were randomized into the treatment group which received an SDM pamphlet and standardized discussion by one of 3 providers (LF, BK, MD). The control group did not receive the SDM pamphlet and underwent routine counseling from the treating Mohs surgeon (Fig 2). All enlisted patients completed the SDMQ9 survey at the time of their appointment. The SDMQ9 survey is a validated, 9-item self-assessment that quantifies the patient’s understanding of their role in decision making (Fig 1). The presence or absence of SSI was recorded and monitored for up to 1 month in all patients. SSI was defined by the Centers for Disease Control and Prevention criteria as the following: the presence of characteristic clinical signs of infection at the surgical site (pain, tenderness, warmth, erythema, purulent drainage) as diagnosed by the Mohs surgeon. For all eligible patients, additional parameters were collected, including patient demographics (age, gender), date of procedure, anatomic location of procedure, number of Mohs stages required, final defect size (cm squared), wound repair type, antibiotic administration (none, pre-operative, postoperative, or both), antibiotic type, and epidermal suture technique. The primary outcome of this study is patients’ satisfaction according to the SDMQ9 survey (Fig 1). Secondary outcomes include the rate of SSI as defined by the Centers for Disease Control, and the rate of AP selection in the group with SDM compared to the group without SDM.

### Statistical analysis

The data consist of patient responses to the SDMQ9 survey, a validated tool. Demographic variables, tumor characteristics, surgical characteristics, and the presence or absence of postoperative SSI were collected and recorded. The analysis evaluates the individual items on the SDMQ9 tool and the total score overall and between the groups. In preliminary analyses, numeric variables are compared using a t-test or Mann–Whitney U test (due to non-normality) and categorical variables are compared by chi-square tests or Fishers Exact test. We assessed the reliability and validity of the SDMQ9 in our sample using exploratory factor analysis employing principal component analysis. Exploratory logistic regressions analyzed the relationship of the primary outcome, whether the patient chose antibiotic prophylaxis or not, by predictor variables significant in preliminary analyses. All analyses were conducted using R and SPSS v28 where two-sided *P*-values less than .05 were considered statistically significant.

## Results

### Demographics

Of the 480 total cases of MMS performed at Scripps Clinic Bighorn Mohs Surgery Center during the timeframe listed, 51 (10%) met full inclusion criteria. Age, sex, size of defect, number of Mohs stages, tumor type, and wound repair type were found to be similar between groups (Table I).

**Table I:** Demographics, surgical qualitative information, and SDMQ9 scoring for the 51 patients meeting inclusion criteria of the selected time period

	Shared Decision-Making Group (*n* = 25)	Placebo Group (*n* = 26)	*P*-value
Gender			.121
Male	9 (36.0%)	15 (57.7%)	
Female	16 (64.0%)	11 (42.3%)	
Average age			
Mean (SD)	75.5 (9.2)	74.4 (7.25)	.626
Median (IQR)	75 (16)	74.5 (10)	.902
Tumor type			.755
SCC	18 (72%)	20 (76.9%)	
BCC	7 (28%)	6 (23.1%)	
Average Mohs stages	1.3 (range 1–5)	1.2 (range 1-2)	.660
Average defect size (cm^2^)			
Mean (SD)	4.4 (2.2)	3.9 (2.7)	.54
Median (IQR)	3.9 (2.7)	2.8 (3.3)	.21
Repair type			.867
Secondary intention	2 (8%)	1 (3.8%)	
Primary linear closure	22 (88%)	24 (92.3%)	
Xenograft	1 (4%)	1 (3.8%)	
Management decision			.061
Observation	20 (80%)	13 (50.0%)	
Preoperative antibiotic	0 (0%)	3 (11.5%)	
Postoperative antibiotic	5 (20%)	9 (34.6%)	
Both Pre- and post-operative antibiotic	0 (0%)	1 (3.8%)	
Antibiotic used			.025
Yes	5 (20%)	13 (50%)	
No	20 (80%)	13 (50%)	
Antibiotic choice			.200
Cephalexin	0	5	
Doxycycline	5	8	
Rate of postoperative surgical site infection	2/25 (8%)	2/26 (7.7%)	.668
SDMQ9 score			
Mean (SD)	4.732 (0.4259)	2.1846 (1.820)	<.001
Median (IQR)	5 (0.5)	2 (3.475)	<.001

In the group that received the SDM pamphlet and discussion, 16 were female and 9 were male. The average age was 75.5 years old (SD 9.2). The average number of Mohs stages required to clear the defect was 1.3 (range 1-5). The average defect size was 4.4 cm^2^. Repair types included 22 primary linear closure (PLC), 1 xenograft, and 2 defects undergoing secondary intention.

In the control group that did not receive the SDM pamphlet and discussion, 11 were female and 15 were male. The average age was 74.4 years old (SD 7.25). The average number of Mohs stages required to clear the defect was 1.2 (range 1-2). The average defect size was 3.9 cm^2^. Repair types included 17 PLC, 1 xenograft, and 1 secondary intention.

### Between group differences

There were higher rates of antibiotic use in the control group (50% vs 20%, *P* = .025). The SDMQ9 variable was not normally distributed; therefore, Mann–Whitney U test was used, median and IQR were reported. The median SDMQ9 Score was higher in the treatment group [5 (0.5) vs 2 (3.475), *P* < .001].

#### Reliability

Full Data Together (*n* = 51): our sample KMO = 0.858, *P* < .001; thus, factor analysis is suitable. The inter-item correlation is 0.896 and Cronbach's alpha for the whole dataset (treatment and control, *n* = 51) is 0.987 (0.987 standardized), which indicates a high level of internal consistency and reliability for the tool with this specific sample of 51 participants. All the items are moderately to highly correlated (0.8–0.99). The removal of any one item would not help the scale mean improve, in the item-totals.

In the whole group (*n* = 51), we see in the communalities that 1 component was extracted and it explains 88.2% of the variance in q1, 93.2% of the variance in q2, etc. In the total variance explained, we see that the 1 component accounts for/explains 90.81% of the variance in all 9 items. This is a significant portion of variance, and no other components were extracted to load and account for a higher variance explained. In the component matrix, we see that only 1 component was extracted (all eigen values were greater than 1), all the item load to 1 component as they all have a number greater than 0.3, and no rotation is needed if there is 1 extraction. This information leads us to believe that no item needs to be removed from the scale to make the tool have greater validity. Overall, the tool for our sample was reliable and valid as a whole and in the control group.

## Power

Power analyses are completed posthoc.

### Any antibiotic use

As this variable is categorical and was assessed using a chi-square test, the family of tests for the family will be chi-square, and we will assess as a goodness of fit (the results are similar for generic tests). The effect size is 0.60, alpha (error) of 0.05, sample size of 51, and degrees of freedom is 1. With these parameters, the noncentrality parameter is 18.36, critical χ2 is 3.841, and power achieved is 99%. For those who received SDM counseling, 20% were prescribed antibiotics in comparison to 50% of patients in the control group (*P* = .025).

### Management decision

In the SDM treatment group, 20 patients (80%) chose to observe and defer AP, 0 (0%) chose preoperative AP, 5 (20%) chose postoperative AP. In the placebo group, 13 patients (50%) chose to observe and defer AP, 3 (11.5%) chose preoperative AP, 9 (34.6%) chose postoperative AP and 1 (3.8%) chose both pre- and postoperative AP. Management decision trended toward significance, *P* = .061.

### Rate of postoperative surgical site infection

The number of patients prescribed any form of antibiotic prophylaxis (either preoperative or postoperative) differed between the 2 treatment groups. Twenty percent of patients in the SDM treatment group were given antibiotics while 50% of patients in the placebo group were given antibiotics. Of note, while more patients in the placebo group were prescribed antibiotics, the rate of postoperative SSI was similar (7.7% versus 8%, *P* = .668).

### SDMQ9 Score

The SDMQ9 is a standardized 9-item self-assessment that quantifies the patient’s understanding of their role in decision making and has been validated in multiple clinical studies^18^^,^^20^ (Fig 1). A numerical score was applied for each answer choice as follows: (0) completely disagree, (1) strongly disagree, (2) somewhat disagree, (3) somewhat agree, (4) strongly agree, (5) completely agree. The numbers corresponding to each answer were then averaged together. As this variable is numeric and assessed using a nonparametric t-test, the Mann–Whitney U test, the family’s t-tests and testing means under the Mann–Whitney test, 2-tailed, normal parent family, with effect size of 1.91, alpha of 0.05, and sample size of 25 and 26. The noncentrality parameter is 6.663, Critical t is 2.01, degrees of freedom is 46.7, and achieved power of 99%. Overall, the study is well powered.

The average SDMQ9 score in the SDM treatment group was 4.73 versus 2.18 in the placebo group (*P* < .001).

## Discussion

Shared decision-making tools have been effective in helping physicians educate patients about their specific disease and management options.^21^ Both physicians and patients have found SDM to be helpful and effective in their decision-making process and SDM may improve the quality of care for patients.^21^^,^^23^ SDM tools have been implemented in various aspects of medical dermatology; however, to our knowledge, this is the first use of SDM in dermatologic surgery.^23^ In the absence of medical risk factors (prosthetic heart valve, prosthetic joint replacements, diabetes mellitus, peripheral arterial disease, tobacco use, etc.), the effectiveness of AP in reducing SSI in dermatologic surgery remains controversial. Physicians are cognizant and intentional about their use of antibiotics considering the growing evidence of antibiotic resistance and multiple potential side effects of taking oral antibiotics.^17^^,^^18^ In light of the evidence that AP does not reduce the risk of SSI following MMS on the lower extremities,^1^^,^^6^^,^^11^ our study aimed to evaluate the role in SDM in patient satisfaction and in guiding management decisions concerning the use of AP.

In the study presented above, we found that the rate of AP use in those that received a formal discussion of SDM was lower (20% of patients in SDM treatment group compared to 50% of patients in placebo group, *P* = .025). Despite this lower usage of AP to theoretically prevent infection, the rate of postoperative SSI was similar (8% in SDM treatment group versus 7.7% in control group, *P* .668). This aligns with previous studies that failed to demonstrate that AP reduces the risk of SSI.^1^^,^^6^^,^^11^ Interestingly, of the 4 patients within both treatment and control groups, half of them received AP.

Our results demonstrate higher patient satisfaction scores measured by the SDMQ9 in the SDM treatment group, which was statistically significant (4.73 compared to 2.18, *P* < .001). This illustrates that patients found the SDM pamphlet to be effective in helping them understand their medical condition, their risk of SSI following MMS, and their management options. As physicians often find themselves with inadequate time to meet demands of a high-volume practice, we recognize that the additional time required for SDM must be justified. The improvement in patient satisfaction scores through presentation of a SDM pamphlet suggests that patients feel a higher degree of partnership and trust with their physician which may have a wide and positive impact. It may enable patients to be more forthcoming about concerns and symptoms and, by having them trust in their physician’s commitment to their best interests, both better outcomes and reduced malpractice issues may be achieved. In addition, because each patient comes with a different life experience, life demands, and core values, the optimal decision may be different for different patients; this appropriate decision can be reached by incorporating the patient-specific realities. Otherwise, applying random or one-size-fits-all decisions may miss the mark for many patients. We believe that these potential benefits justify the increased use of SDM in this setting. In future studies, it would be beneficial to analyze the reasons that guide each patient’s management selection, as this may serve to direct specific aspects of patient education.

## Conclusion

The utility of antibiotic prophylaxis following MMS on the lower extremities in the absence of confounding conditions remains controversial in the literature. This study aims to explore the best ways to educate patients about the risk of SSI following MMS on the lower extremities, the lack of evidence of risk reduction with AP, and management options following their surgery. SDM is a useful educational tool used in many specialties to guide patient education and management discussion. In the study presented above, we were able to demonstrate that the use of SDM was associated with higher patient satisfaction scores. We also found that the use of SDM resulted in lower rates of AP prescription, while maintaining an equivalent rate of SSI. This study helps explore the role of SDM in dermatologic surgery with the goal of reducing SSI following MMS and improving patient outcomes.

## Conflicts of interest

None disclosed.
